# Assessment of Clinical Usefulness of Resting Electrocardiogram (PH-ECG Score) in Monitoring the Efficacy of Balloon Pulmonary Angioplasty (BPA) in Patients with Chronic Thromboembolic Pulmonary Hypertension (CTEPH)

**DOI:** 10.3390/jcm10194548

**Published:** 2021-09-30

**Authors:** Michał Piłka, Małgorzata Mańczak, Szymon Darocha, Marta Banaszkiewicz, Rafał Mańczak, Michał Florczyk, Piotr Kędzierski, Arkadiusz Pietrasik, Paweł Balsam, Paweł Kurzyna, Marcin Wasilewski, Rafał Wolański, Adam Torbicki, Marcin Kurzyna

**Affiliations:** 1 Department of Pulmonary Circulation, Thromboembolic Diseases and Cardiology, Centre of Postgraduate Medical Education, European Health Centre Otwock, 05-400 Otwock, Poland; szymon.darocha@ecz-otwock.pl (S.D.); marta.banaszkiewicz@ecz-otwock.pl (M.B.); rafal.manczak@ecz-otwock.pl (R.M.); michal.florczyk@ecz-otwock.pl (M.F.); piotr.kedzierski@ecz-otwock.pl (P.K.); adam.torbicki@ecz-otwock.pl (A.T.); marcin.kurzyna@ecz-otwock.pl (M.K.); 2 Department of Gerontology, Public Health and Didactics National Institute of Geriatrics, Rheumatology and Rehabilitation, 02-637 Warsaw, Poland; m.manczak@op.pl; 3 1st Chair and Department of Cardiology, Medical University of Warsaw, 02-091 Warsaw, Poland; apietrasik@tlen.pl (A.P.); pawel.balsam@me.com (P.B.); paw.kurzyna@gmail.com (P.K.); marcin.vasilewski@wp.pl (M.W.); rafalwolanski@gmail.com (R.W.)

**Keywords:** PH-ECG score, balloon pulmonary angioplasty, chronic thromboembolic pulmonary hypertension, electrocardiography

## Abstract

Background: Balloon pulmonary angioplasty (BPA) is a form of therapy for chronic thromboembolic pulmonary hypertension (CTEPH). The study objective is to assess the clinical usefulness of resting ECG (PH-ECG score) in monitoring the efficacy of BPA in CTEPH patients. Methods and results: Ninety-four (*n* = 94) CTEPH patients were included in the analysis. A standard 12-lead-ECG was performed before the first BPA session and after completion of treatment. The whole analysed population (*n* = 94) was divided into the following two groups: derivation cohort (*n* = 41) and validation cohort (*n* = 53). The derivation cohort was divided into the following two subgroups: patients with mean pulmonary artery pressure (mPAP) after the completion of therapy < 25 mmHg (*n* = 21) and patients with mPAP after the completion of therapy ≥ 25 mmHg (*n* = 20). In the first subgroup, four (R-wave V1 + S-wave V5/V6 > 10.5 mm, QRS-wave axis > 110 degrees, R-wave V1 > S-wave V1, SIQIII pattern) of the six ECG parameters of overload of the right cardiac chambers showed statistically significant differences (*p* < 0.005). That was followed by a determination of the sensitivity and specificity, positive (PPV) and negative predictive value (NPV), and ROC curve (AUC 0.9; 95% CI: 0.792–1.000) for the variable that was a sum of the above four ECG parameters (PH-ECG score). The absence of all of the four ECG parameters at rest (PH-ECG score = 0) well reflected patients with mPAP < 25 mmHg (sensitivity, 100%; specificity, 80%; PPV, 84%; NPV, 100%). In the validation cohort with mPAP < 25 mmHg and PH-ECG score = 0, sensitivity, specificity, PPV, and NPV were 86%, 77%, 73%, and 89%, respectively. Conclusions: Resting ECG trace is clinically useful in the monitoring of therapeutical effects of BPA in CTEPH patients.

## 1. Introduction

Chronic thromboembolic pulmonary hypertension (CTEPH) is a form of pulmonary hypertension characterised by the presence of chronic embolic material in the pulmonary arteries, which persists despite at least three months of effective therapy with antithrombotic agents and progressive remodelling of the other patent pulmonary vessels [1]. Currently, there are several available CTEPH therapies. The basic type of therapy, if the patient’s condition and the morphology of thromboembolic lesions allows it, is pulmonary endarterectomy (PEA) [2,3,4,5]. Many CTEPH patients, however, are not qualified for PEA due to the extreme peripheral location of thrombi in the pulmonary arteries, which makes it impossible for the surgeon to reach them, or due to severe comorbidities. Such patients may be subjected to a series of balloon pulmonary angioplasty (BPA) procedures [6,7,8,9,10]. An assessment of the effects of subsequent BPA sessions, as well as long-term follow-up of patients after the completed therapy, requires, for example, invasive examination, such as right heart catheterization (RHC) [11]. The main purpose of this study is to create a simple electrocardiographic (ECG) scale, useful in clinical practice, which could be used to monitor the efficacy of interventional therapies, such as BPA, in patients with CTEPH.

## 2. Materials and Methods

The analysis included CTEPH patients hospitalised at the European Health Centre, Otwock, Poland between 2013 and 2021. All the patients included in the study were diagnosed with CTEPH and qualified for interventional treatment with BPA in accordance with the standards of the European Society of Cardiology [5,12].

The study group was divided into the following two cohorts: the derivation cohort and the validation cohort. From the derivation cohort we extracted the ECG parameters that were important to predict a final mPAP < 25 mmHg after BPA therapy; in the validation cohort, the validity of these parameters was assessed. The first 41 patients, who completed the BPA therapy between 2013 and 2016, were included in the derivation cohort and the remaining 53 patients, hospitalized between 2017 and 2021, constituted the validation cohort.

The baseline ECG, six-minute walk test (6MWT), and laboratory tests were performed 1 day before the first BPA and a control evaluation (RHC, 6MWT, ECG, laboratory tests) was conducted 3 to 6 months after the last BPA procedure. Baseline haemodynamics (RHC) were evaluated just before the first BPA session.

Patients with significant cardiac arrhythmias, such as atrial fibrillation, or significant ECG changes secondary to coronary heart disease due to difficulties in the analysis of the ECG curve were excluded from the analysis. The institutional ethics committee approved the study protocol (decision number 88/PB/2015). All the patients agreed to participate in the study.

### 2.1. Right Heart Catheterization and Balloon Pulmonary Angioplasty

Right heart catheterization was performed via the internal jugular vein or femoral vein in accordance with applicable standards [13]. The following haemodynamic parameters were measured or calculated: mean right atrial pressure (RAP), systolic pulmonary artery pressure (sPAP), mean pulmonary artery pressure (mPAP), pulmonary vascular resistance (PVR), and cardiac index (CI). The mPAP in all patients was at least 25 mmHg, and the pulmonary capillary wedge pressure (PCWP) was lower than or equal to 15 mmHg. Treatment by BPA was performed by two experienced interventional cardiologists in accordance with the established treatment protocol.

### 2.2. Six-Minute Walk and Laboratory Tests

The six-minute walk test (6MWT) was performed by qualified medical personnel in accordance with applicable standards of the American Society of Cardiology [14]. The degree of myocardial necrosis was assessed by measurement of the ultra-sensitive troponin level (Roche, Mannheim, Germany; plasma, normal values < 0.003 ng/mL), and the severity of cardiac failure by measurement of the NTproBNP level (Roche, Mannheim, Germany; serum, normal values < 125 pg/mL).

### 2.3. Electrocardiogram

**Figure 1:**
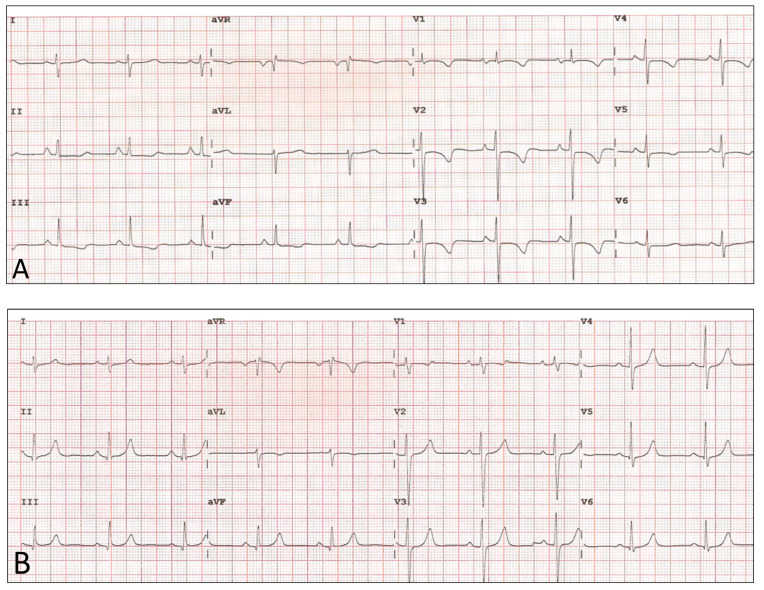
Electrocardiogram before (**A**) and after (**B**) 5 sessions of BPA—27-year-old female with mPAP 49 mmHg (**A**) before therapy and 24 mmHg (**B**) after therapy.

For all patients, the standard 12-lead ECG was performed in the supine position with a commercially available ECG system (Philips PageWriter TC50, Andover, MA, USA; paper speed was 25 mm/s; 1 mV = 10 mm). Baseline ECG was performed one day before BPA, and a follow-up examination 3 to 6 months after the last BPA session (Figure 1). The following electrocardiographic parameters of hypertrophy and overload of the right heart were analysed: P wave amplitude II > 2.5 mm, R wave amplitude V1 + S wave amplitude V5/V6 > 10.5 mm, QRS wave axis > 110 degrees, R wave amplitude V1 > S wave amplitude V1, RBBB (Right Bundle Branch Block), and SIQIII pattern.

### 2.4. Statistical Analysis

Statistical analysis was performed with Statistica PL software (version 13, STATSOFT, Tulsa, OK, USA). Continuous variables with normal distribution were presented as means and standard deviations, while those that were not normally distributed were presented as medians and interquartile ranges. Dependent Student’s *t*-test or Wilcoxon test was applied to compare the continuous variables before and after interventional treatment with BPA (depending on the distribution of the analysed variable assessed using the Shapiro–Wilk test). McNemar’s test was used to compare nominal variables. Mann–Whitney U or Student’s *t*-test and chi-square tests were applied to compare baseline parameters in both study groups (derivation and validation cohort). The sensitivity and specificity, positive and negative predictive values, and the ROC curve of the PH-ECG score, which was defined as the sum of four statistically significant ECG parameters of hypertrophy and overload of the right heart, were determined. Statistical significance in the study was established at *p* < 0.05.

## 3. Results

The ECG results of the 109 CTEPH patients treated with BPA were analysed. Fifteen patients dropped out of the study because their ECG curves could not be interpreted (stimulator, arrhythmia, death before the end of treatment). Finally, ninety-four (*n* = 94) patients were included in the study. The entire population of 94 patients was divided into the following two groups: the derivation cohort (*n* = 41) and the validation cohort (*n* = 53). The patients in the derivation cohort had higher concentrations of NTproBNP. Even so, no statistically significant differences were found in the values of the hemodynamic and functional parameters between both groups of patients before the BPA procedure. General characteristics and comparison of baseline parameters for both groups of patients enrolled in the study are presented in Table 1.

The derivation cohort was divided into the following two subgroups: patients with mPAP after completion of therapy < 25 mmHg (*n* = 21) and patients with mPAP after completion of therapy ≥ 25 mmHg (*n* = 20). From the subgroup comprised of the patients who, after their interventional therapy with BPA, had mPAP below 25 mmHg (*n* = 21), we extracted the statistically significant ECG parameters. Table 2 compares the functional, haemodynamic, and ECG parameters in the subgroup of patients with mPAP below 25 mmHg after BPA therapy.

In the subgroup of patients with a very good haemodynamic effect after BPA (mPAP < 25 mmHg), four statistically significant (R wave amplitude V1 + S wave amplitude V5/V6 > 10.5 mm, QRS wave axis > 110 degrees, R wave amplitude V1 > S wave amplitude V1, SIQIII pattern) differences were observed regarding the six ECG parameters of overload of the right cardiac chambers. Subsequent measurements included the sensitivity, specificity, and positive and negative predictive values for the variable being a sum of four statistically significant ECG parameters (PH-ECG score) observed in the subgroup of patients with mean pulmonary artery pressure below 25 mmHg after BPA (Table 3). The absence of all of the four ECG parameters at rest (PH-ECG score = 0) was observed in the patients with mPAP < 25 mmHg (sensitivity, 100%; specificity, 80%; positive predictive value, 84%; negative predictive value, 100%).

**Figure 2:**
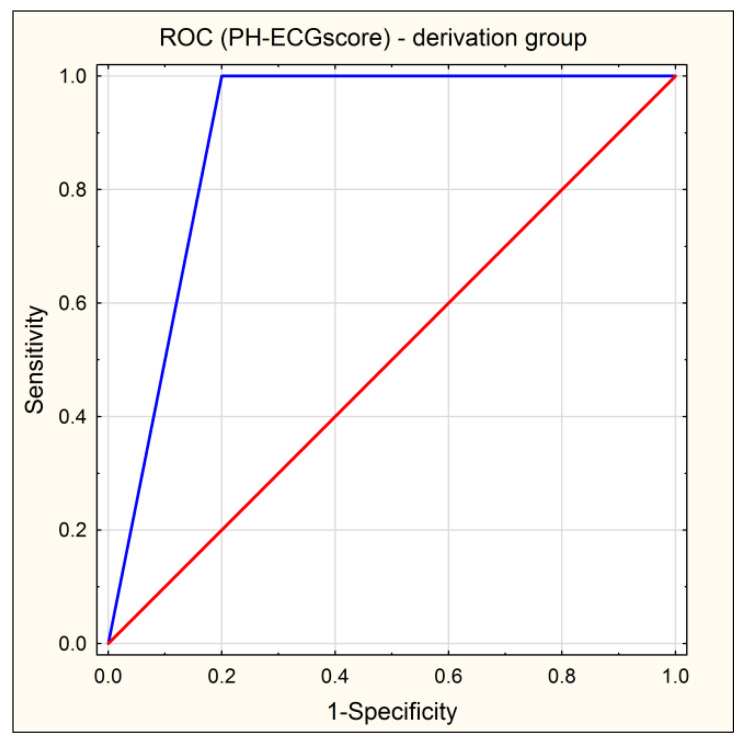
ROC curve of the PH-ECG score (blue line), which is the sum of the four statistically significant ECG parameters in patients who underwent BPA. Red line indicates reference line.

The AUC of the ROC curve of PH-ECG score = 0 was 0.900 (95% CI: 0.792–1.000) (Figure 2).

Table 4 compares the haemodynamic and functional parameters in the validation cohort.

Table 5 compares the functional, hemodynamic, and ECG parameters of the validation cohort in the patients with mPAP < 25 mmHg after BPA therapy.

In the validation cohort with mPAP < 25 mmHg and PH-ECG score = 0, the sensitivity, specificity, PPV, and NPV were 86%, 77%, 73%, and 89%, respectively (Table 6).

**Figure 3:**
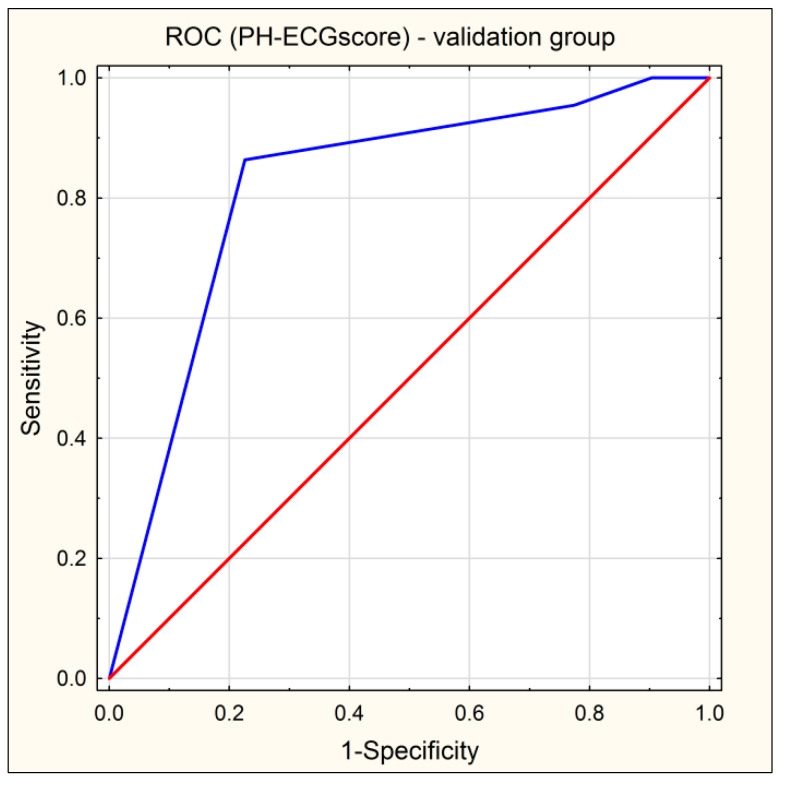
ROC curve of PH-ECG score (blue line) in the validation cohort. Red line indicates reference line.

The AUC of the ROC curve of PH-ECG score = 0 in the validation cohort was 0.819 (95% CI: 0.699–0.939) (Figure 3).

## 4. Discussion

Pulmonary hypertension, regardless of its aetiology, leads to the progressive failure of the right ventricle and, consequently, to death. Significant haemodynamic decompression of the right ventricle, leading to improvement in its function, is an important predictive factor for the survival of patients with pulmonary hypertension, as already confirmed in a study by Rich et al. twenty-eight years ago, which showed that a good response to treatment and resulting improvement in haemodynamic parameters were significantly correlated with the survival of patients with pulmonary hypertension [15].

Progressive right ventricular heart failure is correlated with certain changes in the ECG curve—the so-called ECG signs of hypertrophy and overload of the right ventricle and right atrium [16,17,18].

The prognostic value of selected ECG parameters was confirmed in a few studies on patients with pulmonary hypertension [19,20,21].

Several previous studies have shown that regression of specific ECG signs of right ventricular and right atrial hypertrophy and overload is observed in patients with CTEPH after a successful procedure of PEA, and in patients with pulmonary arterial hypertension treated with specific pharmacotherapy [22,23,24].

It has been shown that, after PEA, a change in the P wave amplitude in lead II, in the R wave amplitude in lead V1, and in the number of patients with a negative T wave from V1 to V3 is observed in the first month of the follow-up, with no further changes in the course of the 12-month follow-up. A change in the S amplitude in V1, in the R:S wave amplitude ratio in V6, and in the SIQIII complex was observed during the 12 months of follow-up [23].

The use of BPA for CTEPH treatment resulted in a significant improvement in the prognosis for patients who, for various reasons, could not undergo cardiosurgical treatment (PEA) [25]. Currently, thanks to improvements in the BPA method, the mPAP may be normalised in a significant number of patients with CTEPH [8].

Patients who complete the BPA therapy require periodic, non-invasive control, as well as RHC. In previous studies, it has been documented that BPA therapy and an improvement in haemodynamic parameters are associated with significant changes in the resting ECG [26,27,28,29].

Our study is the first to adopt an easy-to-use electrocardiographic scale (PH-ECG score) to quantify the usefulness of a resting ECG in monitoring the effects of CTEPH patients treated with BPA.

A study by Nishiyama et al. revealed that the treatment of CTEPH patients with BPA caused a withdrawal of certain ECG signs of right ventricular and right atrial hypertrophy and overload, which manifested as a significant improvement in the right ventricular systolic function. The ROC analysis in this study confirmed that the value of the S wave amplitude in lead V5, the sum of the R wave amplitude in lead V1 and the S wave amplitude in lead V5, the S wave amplitude in lead I, and the QRS axis were important predictors of mPAP ≥ 30 mmHg. Additionally, following BPA, a significant correlation was observed between the change in mPAP and changes in the values of the following ECG parameters: S wave in lead I, sum of amplitude of R wave in lead V1 and S wave in lead V5, and P wave amplitude in lead II. The study also showed that an R:S wave amplitude ratio in lead V1 ≥ 1 might be more frequently observed in CTEPH patients whose haemodynamic parameters improved following treatment with BPA [26].

Electrocardiographic signs of right heart hypertrophy and overload during BPA treatment were also analysed in a study by Yokokawa T et al. The study assessed the ECG criteria of right ventricular hypertrophy and overload, according to American recommendations, in 19 CTEPH patients after subsequent BPA sessions. Out of the fifteen ECG criteria, the S wave amplitude in lead V6 (*p* = 0.005) and (the maximum R wave amplitude in V1,2 + the maximum S wave amplitude in I, aVL—the S wave amplitude in V1) (*p* = 0.046) changed significantly after interventional treatment with BPA. The mean number of ECG criteria of right heart hypertrophy and overload observed after intervention significantly reduced (4.8 ± 2.6 vs. 3.1 ± 2.5; *p* = 0.003) [28].

In our recent study, we showed that haemodynamically effective BPA, defined as a decrease in the PVR of above 49%, resulted in a significant change in the values of the following electrocardiographic parameters: T wave axis, P wave amplitude in lead II, S wave amplitude in lead V5, and R:S amplitude ratio in lead V5. In the group of patients with an inferior response, there were no significant changes in the above parameters. In the whole analysed population, the percentage change in the PVR was significantly correlated with the percentage change in the values of the following ECG parameters: axis of the QRS (rho = 0.530) and T wave (rho = 0.372), *P* wave amplitude in leads II (rho = 0.340) and III (rho = 0.430), S wave amplitude in lead V_5_ (rho = 0.634), R/S amplitude ratio in lead V_5_ (rho = –0.636), S wave amplitude in lead V_6_ (rho = 0.508), and S wave amplitude in lead I (rho = 0.496) [27].

The present study is the first to validate a simple ECG scale (PH-ECG score) consisting of four selected ECG parameters (R wave amplitude V1 + S wave amplitude V5/V6 > 10.5 mm, QRS wave axis > 110 degrees, R wave amplitude V1 > S wave amplitude V1, SIQIII pattern), which may serve as an additional tool when evaluating if a patient is having a good evolution with BPA therapy, but together with functional data, right ventricle remodelling on echocardiography, and clinical and biomarker parameters. Thanks to the above, the widely available non-invasive examination, in the form of a resting ECG, may help with deciding on when to terminate the therapy and assess the long-term effects of BPA.

### Study Limitations

The first limitation of the study is that the group of CTEPH patients who underwent BPA was moderately large. The second limitation is the lack of analysis of changes in the morphology and function of the right heart assessed by imaging examinations (such as NMR and ECHO) in relation to the ECG curve.

## 5. Conclusions

In CTEPH patients treated with BPA, a simple scale (PH-ECG score), based on the analysis of changes in the ECG curve, may serve as an additional tool for deciding on when to terminate the therapy and assess the long-term effects of BPA.

## Figures and Tables

**Table 1:** General characteristics and comparison of baseline parameters for both groups of patients (derivation and validation cohort) subjected to percutaneous vascular intervention (BPA).

General Characteristics of the Population	Derivation Cohort (*n* = 41)	Validation Cohort (*n* = 53)	*p*
Female sex, *n* (%)	19 (46.4%)	23 (43.4%)	0.87
Age, y, mean (SD)	57.17 (17.12)	61.02 (14.43)	0.6
WHO functional class, *n* (%)			0.54
I	*n* = 1 (2.4%)	*n* = 1 (1.9%)	
II	*n* = 5 (12.3%)	*n* = 14 (26.4%)	
III	*n* = 29 (70.7%)	*n* = 36 (67.9%)	
IV	*n* = 6 (14.6%)	*n* = 2 (3.8%)	
NTproBNP (pg/mL), median (IQR)	1377 (339.3–3683)	510.9 (288–1512)	<0.001
Troponin (pg/mL), median (IQR)	0.011 (0.006–0.019)	0.012 (0.007–0.018)	0.43
6MWT (m), mean (SD)	349.7 (167.94)	391.8 (114.31)	0.07
mPAP (mmHg), mean (SD)	48.17 (11.68)	45.3 (9.37)	0.07

**Table 2:** Comparison of haemodynamic, functional, and electrocardiographic parameters in patients with mPAP < 25 mmHg (*n* = 21) after BPA.

Haemodynamic Parameters	before BPA	after BPA	*p*-Value
RAP, mmHg, median (IQR)	8 (5–10)	3 (2–6)	0.006
PAPs, mmHg, mean (SD)	73.29 (20.16)	35.95 (4.73)	<0.001
PAPm, mmHg, mean (SD)	44.24 (11.29)	21.1 (2.26)	<0.001
PVR, Wood units, mean (SD)	8.72 (4.36)	2.63 (0.67)	<0.001
CI, l/min/m^2^, mean (SD)	2.57 (0.79)	2.78 (0.56)	0.24
**Functional Parameters**			
NTproBNP, pg/mL, median (IQR)	981.4 (329.9–2535)	102 (50.4–186.6)	<0.001
WHO FC, *n*, median (IQR)	3 (3–3)	1 (1–2)	0.002
6MWT, m, mean (SD)	390.52 (157.54)	496.62 (120.89)	<0.001
Troponin, ng/mL, median (IQR)	0.011 (0.006–0.015)	0.006 (0.006–0.013)	0.07
**Electrocardiographic Parameters**			
P wave amplitude II > 2.5 mm, *n* (%)	5 (23.8%)	0	0.063
R wave amplitude V1 + S wave amplitude V5/V6 > 10.5 mm, *n* (%)	9 (42.9%)	0	0.004
QRS wave axis > 110 degrees, *n* (%)	6 (28.6%)	0	0.031
R wave amplitude V1 > S wave amplitude V1, *n* (%)	7 (33.3%)	0	0.016
RBBB (Right Bundle Branch Block), *n* (%)	4 (19.0%)	3 (14.3%)	1
SIQIII pattern, *n* (%)	8 (38.1%)	0	0.008

**Table 3:** Sensitivity, specificity, and positive and negative predictive values of the PH-ECG score, which is the sum of the four statistically significant ECG parameters in the derivation cohort.

PH-ECG Score (Number of ECG Parameters after Treatment with BPA)	mPAP < 25	mPAP ≥ 25	Sensitivity	Specificity	PPV	NPV
PH-ECG score = 0 (0)	21	4	1.000	0.800	0.840	1.000
PH-ECG score = 1 (1)	0	7	1.000	0.450	0.656	1.000
PH-ECG score = 2 (2)	0	6	1.000	0.150	0.553	1.000
PH-ECG score = 3 (3)	0	3	1.000	0.000	0.512	-
PH-ECG score = 4 (4)	0	0	-	-	-	-

**Table 4:** Comparison of the haemodynamic and functional parameters in the validation cohort (*n* = 53).

Haemodynamic Parameters	before BPA	after BPA	*p*-Value
RAP, mmHg, median (IQR)	8 (6–10)	5 (3–7)	*p* < 0.001
PAPs, mmHg, mean (SD)	79.21 (17.99)	48.22 (16.58)	*p* < 0.001
PAPm, mmHg, mean (SD)	45.32 (9.37)	28.41 (9.66)	*p* < 0.001
PVR, Wood units, mean (SD)	7.42 (3.17)	3.49 (1.94)	*p* < 0.001
CI, l/min/m^2^, median (IQR)	2.73 (2.28–3.18)	2.91 (2.33–3.13)	0.49
**Functional Parameters**			
NTproBNP, pg/mL, median (IQR)	510.9 (288–1512)	171.6 (72.7–533)	0.002
WHO FC, *n*, median (IQR)	3 (2–3)	2 (1–2)	*p* < 0.001
6MWT, m, mean (SD)	391.84 (114.31)	423,39 (125.23)	0.11
Troponin, ng/mL, median (IQR)	0.012 (0.007–0.018)	0.01 (0.006–0.016)	0.65

**Table 5:** Comparison of hemodynamic, functional, and electrocardiographic parameters in patients with mPAP < 25 mmHg (*n* = 22) after BPA (validation cohort).

Haemodynamic Parameters	before BPA	after BPA	*p*-Value
RAP, mmHg, mean (SD)	7.4 (2.45)	3.9 (1.97)	*p* < 0.001
PAPs, mmHg, mean (SD)	73.3 (19.69)	36 (3.66)	*p* < 0.001
PAPm, mmHg, mean (SD)	42.86 (9.68)	21.09 (2.27)	*p* < 0.001
PVR, Wood units, mean (SD)	7.37 (3.96)	2.41 (0.76)	*p* < 0.001
CI, l/min/m^2^, mean (SD)	2.93 (1.11)	2.75 (0.63)	0.26
**Functional Parameters**			
NTproBNP, pg/mL, median (IQR)	334.5 (113.2–1053)	77.65 (48.4–154.5)	0.004
WHO FC, *n*, median (IQR)	3 (2–3)	1 (1–2)	*p* < 0.001
6MWT, m, mean (SD)	401.13 (142.69)	461.28 (134.77)	0.005
Troponin, ng/mL, mean (SD)	0.013 (0.009)	0.008 (0.005)	0.02
**Electrocardiographic Parameters**			
R wave amplitude V1 + S wave amplitude V5/V6 > 10.5 mm, *n* (%)	10 (45.5%)	1 (4.5%)	0.006
QRS wave axis > 110 degrees, *n* (%)	7 (31.8%)	1 (4.5%)	0.001
R wave amplitude V1 > S wave amplitude V1, *n* (%)	9 (40.9%)	1 (4.5%)	0.003
SIQIII pattern, *n* (%)	14 (63.6%)	1 (4.5%)	0.046

**Table 6:** Sensitivity, specificity, and positive and negative predictive values of the PH-ECG score in the validation cohort.

PH-ECG Score (Number of ECG Parameters after Treatment with BPA)	mPAP < 25	mPAP ≥ 25	Sensitivity	Specificity	PPV	NPV	Youden Index
PH-ECG score = 0 (0)	19	7	0.864	0.774	0.731	0.889	0.638
PH-ECG score = 1 (1)	2	17	0.955	0.226	0.467	0.875	0.180
PH-ECG score = 2 (2)	1	4	1.000	0.097	0.440	1.000	0.097
PH-ECG score = 3 (3)	0	2	1.000	0.032	0.423	1.000	0.032
PH-ECG score = 4 (4)	0	1	1.000	0.000	0.415		0.000

## Data Availability

Data available from the authors upon request.
